# Construction and validation of a nomogram for predicting cancer-specific survival in middle-aged patients with advanced hepatocellular carcinoma: A SEER-based study

**DOI:** 10.1097/MD.0000000000039480

**Published:** 2024-09-20

**Authors:** Ziqiang Li, Qingyong Hong, Zhidong Guo, Xiaohong Liu, Chengpeng Tan, Zhe Feng, Kun Li

**Affiliations:** a Department of Hepatobiliary and Pancreatic Surgery, Hubei Provincial Clinical Medicine Research Center for Minimally Invasive Diagnosis and Treatment of Hepatobiliary and Pancreatic Diseases, Zhongnan Hospital of Wuhan University, Wuhan, China; b Department of General Surgery, Tongji Hospital, Tongji University School of Medicine, Shanghai, China.

**Keywords:** advanced hepatocellular carcinoma, cancer-specific survival, middle-aged patients, nomogram, SEER

## Abstract

Hepatocellular carcinoma is the predominant form of primary liver cancer and is the leading cause of cancer-related death. The aim of this study was to construct a nomogram to predict cancer-specific survival (CSS) in middle-aged patients with advanced hepatocellular carcinoma. Clinical data were downloaded from the Surveillance, Epidemiology and End Results (SEER) database for middle-aged patients diagnosed with advanced hepatocellular carcinoma (AJCC stage III and IV) from 2000 to 2019. The patients were randomized in a 7:3 ratio into training cohort and validation cohort. Univariate and multivariate Cox regression analyses were performed in the training cohort to screen for independent risk factors associated with cancer-specific survival for the construction of nomogram. The nomogram was examined and evaluated using the consistency index (C-index), area under the curve (AUC), and calibration plots. The clinical application value of the model was evaluated using decision curve analysis (DCA). A total of 3026 patients were selected, including 2244 in the training cohort and 962 in the validation cohort. Multivariate analysis revealed gender, marital status, American Joint Committee on Cancer (AJCC) stage, tumor size, bone metastasis, lung metastasis, alpha-fetoprotein (AFP) level, surgery, radiotherapy, chemotherapy as independent risk factors, which were all included in the construction of the nomogram. In the training cohort, the AUC values were 0.74 (95% CI: 0.76–0.72), 0.78 (95% CI: 0.82–0.75), and 0.82 (95% CI: 0.86–0.78) at 1-, 3-, and 5-year CSS, respectively. The calibration plots showed good consistency between the actual and predicted values. The DCA curves indicated that the nomogram model could more accurately predict CSS at 1-, 3-, and 5-year in middle-aged patients with advanced hepatocellular carcinoma compared with the AJCC staging system. Highly similar results to the training cohort were also observed in the validation cohort. In the risk stratification system, good differentiation was shown between the 2 groups, and Kaplan–Meier survival analysis indicated that surgery could prolong patient survival. In this study, we developed a nomogram and risk stratification system for predicting CSS in middle-aged patients with advanced hepatocellular carcinoma. The prediction model has good predictive performance and can help clinicians in judging prognosis and clinical decision making.

## 1. Introduction

Primary liver cancer is the sixth most common malignancy and the fourth leading cause of cancer-related death worldwide.^[1]^ The main pathological types of liver cancer include hepatocellular carcinoma (HCC), intrahepatic cholangiocarcinoma and combined hepatocellular-cholangiocarcinoma.^[2]^ Of these, HCC is the most common pathologic type of primary liver cancer, accounting for more than 80% of all types.^[3]^ Because the symptoms of early HCC are often not obvious, approximately 80% of patients are advanced at the time of diagnosis and have a poor prognosis.^[4]^ In addition, most patients with HCC are between 40 and 60 years of age.^[5]^ The prognosis for survival of middle-aged patients remains a very important issue. Surgery is the treatment of choice for early HCC, and studies have shown that patients with early HCC have a 5-year survival rate of 60% after surgical treatment.^[6]^ However, most patients with advanced HCC are lost to surgery and can only be treated with systemic therapy as the preferred treatment option. Sorafenib is an effective first-line drug for the treatment of patients with advanced HCC.^[7]^ Related studies have shown that in addition to severe side effects and eventual drug resistance, the median survival time for patients taking oral sorafenib is only 10 months.^[8]^ The lower survival rate of patients with advanced HCC remains a major problem for clinicians. Therefore, there is a need to develop a prediction model to risk-stratify patients with advanced patients, allowing clinicians to implement more personalized treatment for patients in different strata to improve survival in advanced patients.

Nomogram-based clinical prediction model is a scientifically reliable statistical model that can accurately predict patient survival through a comprehensive analysis of risk factors that affect patient prognosis.^[9]^ Nomogram have been widely used to guide clinical decision-making.^[10–13]^ In fact, many nomogram-based HCC prediction models have been constructed, and in addition prediction models for middle-aged patients with early HCC have been developed and validated.^[5,14,15]^ However, no studies have been conducted to construct nomogram models to predict cancer-specific survival (CSS) for middle-aged patients with advanced HCC.

The aim of this study is to construct a reliable and practical nomogram model for predicting CSS based on important factors obtained from the Surveillance, Epidemiology and End Results (SEER) database to enable clinicians to make better treatment decisions.

## 2. Methods

### 2.1. Patient and variables selection

**Figure 1.**
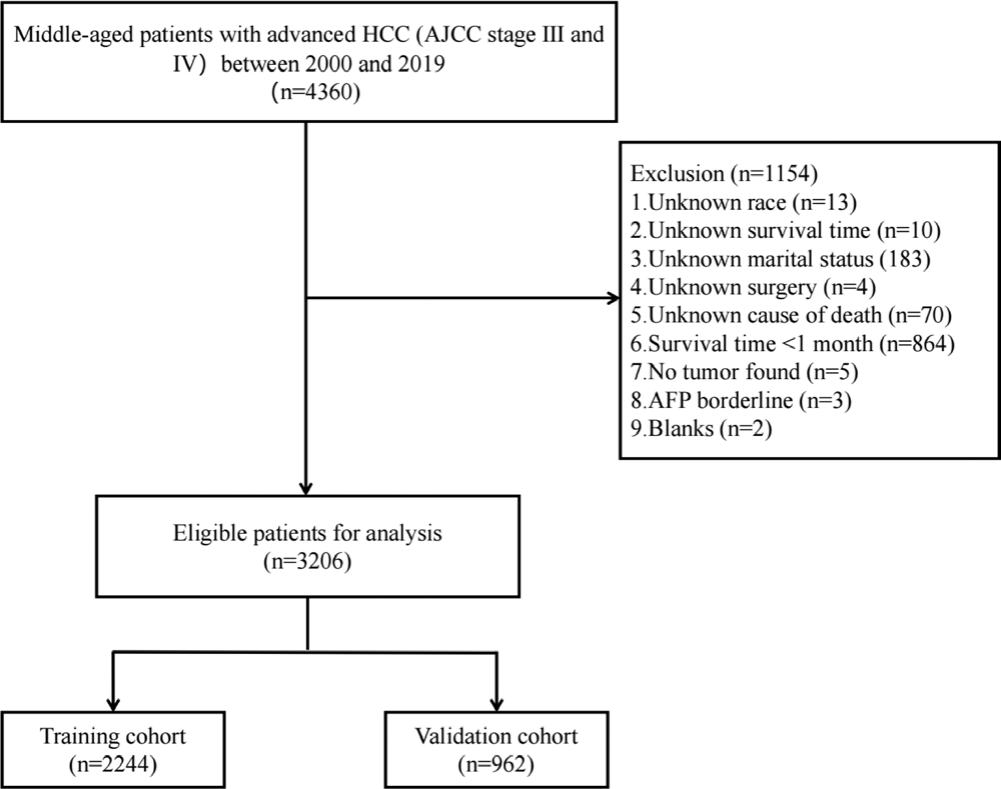
Flow chat of the patient screening. AJCC = American Joint Committee on Cancer, HCC = hepatocellular carcinoma.

Clinical information on HCC patients between 2000 and 2019 was downloaded from the SEER database using SEER*Stat 8.4.0.1 software. Seventeen variables (age, sex, race, marital status, American Joint Committee on Cancer (AJCC) stage, T stage, tumor size, bone metastasis, brain metastasis, lung metastasis, alpha-fetoprotein (AFP), fibrosis score, surgery, radiotherapy, chemotherapy, survival time and survival status) were included, covering demographic characteristics, tumor information, treatment information and survival. Inclusion criteria were as follows: (1) age 40–60 years; (2) International Classification of Diseases for Oncology, Third Edition (ICD-O-3) codes: 8170–8175; (3) patients diagnosed with advanced hepatocellular carcinoma (AJCC stage III and IV). The exclusion criteria were as follows: unknown race and marital status; unknown cause of death; unknown survival time or < 1 month; unknown surgery-related information; AFP borderline (undetermined if positive or negative); and incomplete clinical information. The patient screening process is shown in Figure 1. In addition, we used the 7th edition of the AJCC staging system.

### 2.2. Construction and validation of nomogram

We randomly divided all selected patients into a training cohort (n = 2244) and a validation cohort (n = 962) in a 7:3 ratio. The nomogram model was constructed using the training cohort, and the validation cohort was used for further validation of the prediction model. Univariate analysis and multivariate Cox regression analysis were performed on the training cohort to obtain factors that significantly affected CSS in middle-aged patients with HCC (*P* < .05), and these independent risk factors were used in the construction of the nomogram. The discriminative power of the nomogram was assessed using the C-index and the area under the curve (AUC). The range of C-index and AUC values was 0.5–1.0, where 0.5 indicates random likelihood, and values closer to 1.0 indicate stronger discriminative power, and 1.0 indicates perfect discriminative power of the nomogram. Calibration plots (1000 self-help weight samples) were plotted for 1-, 3- and 5-year to assess the conformity of the model predicted survival with the actual survival. Decision curve analysis (DCA) was used to assess the clinical usefulness of the nomogram. Also, risk scores were assigned to all patients based on the constructed nomogram, and patients were divided into low-risk and high-risk groups based on the best critical values of the model scores. The log-rank test and Kaplan–Meier analysis were used to compare survival differences between groups, and subgroup analysis was performed based on the patients’ surgical informations.

### 2.3. Statistical analysis

Data were extracted from SEER*Stat software (version 8.4.0.1). Categorical variables were expressed as numbers and percentages (n, %), and variables were compared between groups by Chi-square test. Univariate and multivariate Cox regression analyses were performed with SPSS software (version 25.0). Nomogram model was developed and validated using R software (version 4.2.1) and the associated R packages”rms,” “foreign”,” survivor,” “survminer,” “DynNom,” “ggDCA,” and “tableone.” When the *P* value was <.05, it was considered statistically significant.

## 3. Results

### 3.1. Patient characteristics

A total of 3206 eligible middle-aged patients with advanced HCC were enrolled in our study, including 2244 in the training cohort and 962 in the validation cohort. Of these patients, the majority were male (n = 2780, 86.7%), the most common race was white (n = 2125, 66.3%), 1519 (47.4%) were married, 2043 (63.7%) had tumors > 5 cm in size, 383 (11.9%) had bone metastases, 27 (0.8%) had brain metastases, and lung metastases occurred in 454 (14.2%), and AFP-positive patients accounted for the majority (n = 2393, 74.6%). For treatment, the vast majority of patients did not receive surgery (n = 2903, 90.5%), 450 (14.0%) patients received radiotherapy, and 1636 (51.0%) patients received chemotherapy. Table 1 shows the baseline demographic and clinicopathological characteristics of middle-aged patients with advanced HCC, with no significant differences in patient characteristics between the training and validation cohorts.

**Table 1:** Demographics and clinical characteristics of the patients with advanced HCC.

	All cohort	Training cohort	Validation cohort	*P* value
	n = 3206	n = 2244	n = 962	
Age				
40–49	435 (13.6%)	303 (13.5%)	132 (13.7%)	.913
50–59	2771 (86.4%)	1941 (86.5%)	830 (86.3%)	
Sex				
Male	2780 (86.7%)	1930 (86.0%)	850 (88.4%)	.082
Female	426 (13.3%)	314 (14.0%)	112 (11.6%)	
Race				
White	2125 (66.3%)	1487 (66.3%)	638 (66.3%)	.533
Black	529 (16.5%)	362 (16.1%)	167 (17.4%)	
Other	552 (17.2%)	395 (17.6%)	157 (16.3%)	
Marital status				
Married	1519 (47.4%)	1079 (48.1%)	440 (45.7%)	.308
Single	1070 (33.4%)	743 (33.1%)	327 (34.0%)	
Divorced/Separated	537 (16.7%)	362 (16.1%)	175 (18.2%)	
Widowed	80 (2.5%)	60 (2.7%)	20 (2.1%)	
AJCC stages				
III	1596 (49.8%)	1111 (49.5%)	485 (50.4%)	.666
IV	1610 (50.2%)	1133 (50.5%)	477 (49.6%)	
T				
T1	279 (8.7%)	191 (8.5%)	88 (9.1%)	.969
T2	217 (6.8%)	151 (6.7%)	66 (6.9%)	
T3	2129 (66.4%)	1498 (66.8%)	631 (65.6%)	
T4	308 (9.6%)	215 (9.6%)	93 (9.7%)	
Tx	273 (8.5%)	189 (8.4%)	84 (8.7%)	
Tumor size (cm)				
0–5	619 (19.3%)	432 (19.3%)	187 (19.5%)	.815
>5	2043 (63.7%)	1425 (63.5%)	618 (64.2%)	
unknown	544 (17.0%)	387 (17.2%)	157 (16.3%)	
DX bone				
No	2755 (85.9%)	1919 (85.5%)	836 (86.9%)	.586
Yes	383 (11.9%)	276 (12.3%)	107 (11.1%)	
Unknown	68 (2.2%)	49 (2.2%)	19 (2.0%)	
DX brain				
No	3110 (97.0%)	2172 (96.8%)	938 (97.5%)	.099
Yes	27 (0.8%)	24 (1.1%)	3 (0.3%)	
Unknown	69 (2.2%)	48 (2.1%)	21 (2.2%)	
DX lung				
No	2681 (83.6%)	1880 (83.8%)	801 (83.3%)	.884
Yes	454 (14.2%)	316 (14.1%)	138 (14.3%)	
Unknown	71 (2.2%)	48 (2.1%)	23 (2.4%)	
Fibrosis score				
Ishak 0–4	143 (4.5%)	105 (4.7%)	38 (4.0%)	.126
Ishak 5–6	753 (23.5%)	506 (22.5%)	247 (25.7%)	
Unknown	2310 (72.0%)	1633 (72.8%)	677 (70.3%)	
AFP				
Negative	359 (11.2%)	262 (11.7%)	97 (10.1%)	.225
Positive	2393 (74.6%)	1676 (74.7%)	717 (74.5%)	
Unknown	454 (14.2%)	306 (13.6%)	148 (15.4%)	
Surgery				
No surgery	2903 (90.5%)	2026 (90.3%)	877 (91.2%)	.89
Local destruction	106 (3.3%)	76 (3.4%)	30 (3.1%)	
Partial resection	160 (5.0%)	115 (5.1%)	45 (4.7%)	
Transplantation	37 (1.2%)	27 (1.2%)	10 (1.0%)	
Radiation				
No/Unknown	2756 (86.0%)	1927 (85.9%)	829 (86.2%)	.865
Yes	450 (14.0%)	317 (14.1%)	133 (13.8%)	
Chemotherapy				
No/Unknown	1570 (49.0%)	1097 (48.9%)	473 (49.2%)	.914
Yes	1636 (51.0%)	1147 (51.1%)	489 (50.8%)	

AFP = alpha-fetoprotein, AJCC = American Joint Committee on Cancer, HCC = hepatocellular carcinoma.

### 3.2. Univariate and multivariate analysis of CSS

In the training cohort, univariate analysis showed that sex, marital status, AJCC stage, tumor size, T stage, bone metastasis, lung metastasis, AFP level, fibrosis score, surgery, and chemotherapy were risk factors for CSS in patients with HCC (*P* < .05). Multivariate Cox regression analysis showed that sex, marital status, AJCC stage, tumor size, bone metastasis, lung metastasis, AFP level, surgery, radiotherapy, and chemotherapy were independent prognostic factors for CSS (*P* < .05), and they were used in the construction of the nomogram (Table 2).

**Table 2:** Univariate and multivariate Cox regression analyses of CSS in training cohort.

	Univariate		Multivariate	
	HR (95% CI)	*P* value	HR (95% CI)	*P* value
Age				
40–49	Reference			
50–59	1.092 (0.902–1.174)	.667		
Race				
White	Reference			
Black	1.065 (0.941–1.206)	.318		
Other	1.033 (0.917–1.163)	.592		
Sex				
Male	Reference		Reference	
Female	0.814 (0.715–0.928)	.002	0.856(0.750–0.978)	.022
Marital status				
Married	Reference		Reference	
Single	1.283 (1.160–1.419)	<.001	1.124(1.014–1.245)	.025
Divorced/Separated	1.148 (1.010–1.306)	.035	1.057(0.928–1.203)	.405
Widowed	1.041 (0.783–1.384)	.78	0.936(0.702–1.248)	.651
AJCC stages				
III	Reference		Reference	
IV	1.608(1.469–1.760)	<.001	1.242(1.110–1.390)	<.001
T				
T1	Reference			
T2	0.901(0.716–1.133)	.372		
T3	0.887(0.755–1.042)	.144		
T4	1.065(0.864–1.312)	.555		
Tx	1.241(1.002–1.539)	.048		
Tumor size (cm)				
0–5	Reference		Reference	
>5	1.161 (1.032–1.308)	.013	1.257(1.110–1.424)	<.001
Unknown	1.745 (1.503–2.025)	<.001	1.393(1.194–1.625)	<.001
DX bone				
No	Reference		Reference	
Yes	1.589 (1.393–1.812)	<.001	1.416(1.217–1.647)	<.001
Unknown	1.229 (0.907–1.663)	.183	0.612(0.364–1.030)	.064
DX brain				
No	Reference			
Yes	1.255 (0.824–1.911)	.29		
Unknown	1.180 (0.872–1.597)	.283		
DX lung				
No	Reference		Reference	
Yes	1.874 (1.651–2.126)	<.001	1.516(1.317–1.744)	<.001
Unknown	1.365 (1.012–1.842)	.042	1.472(0.880–2.462)	.140
AFP				
Negative	Reference		Reference	
Positive	1.718 (1.482–1.992)	<.001	1.718(1.480–1.995)	<.001
Unknown	1.417 (1.176–1.706)	<.001	1.218(1.007–1.473)	.043
Fibrosis scores				
Ishak 0–4	Reference		Reference	
Ishak 5–6	1.308 (1.040–1.646)	.022	1.160(0.918–1.465)	.215
Unknown	1.459 (1.176–1.810)	.001	1.203(0.966–1.497)	.099
Surgery				
No surgery	Reference		Reference	
Local destruction	0.383 (0.288–0.509)	<.001	0.451(0.339–0.601)	<.001
Partial resection	0.360 (0.287–0.451)	<.001	0.365(0.289–0.462)	<.001
Transplantation	0.167 (0.097–0.289)	<.001	0.210(0.121–0.366)	<.001
Radiation				
No/Unknown	Reference		Reference	
Yes	0.895 (0.789–1.014)	.083	0.719(0.629–0.822)	<.001
Chemotherapy				
No/Unknown	Reference		Reference	
Yes	0.719 (0.658–0.787)	<.001	0.646(0.588–0.709)	<.001

AFP = alpha-fetoprotein, AJCC = American Joint Committee on Cancer, CI = confidence interval, CSS = cancer-specific survival, HR = hazard ratio.

### 3.3. Construction and validation of the nomogram

**Figure 2.**
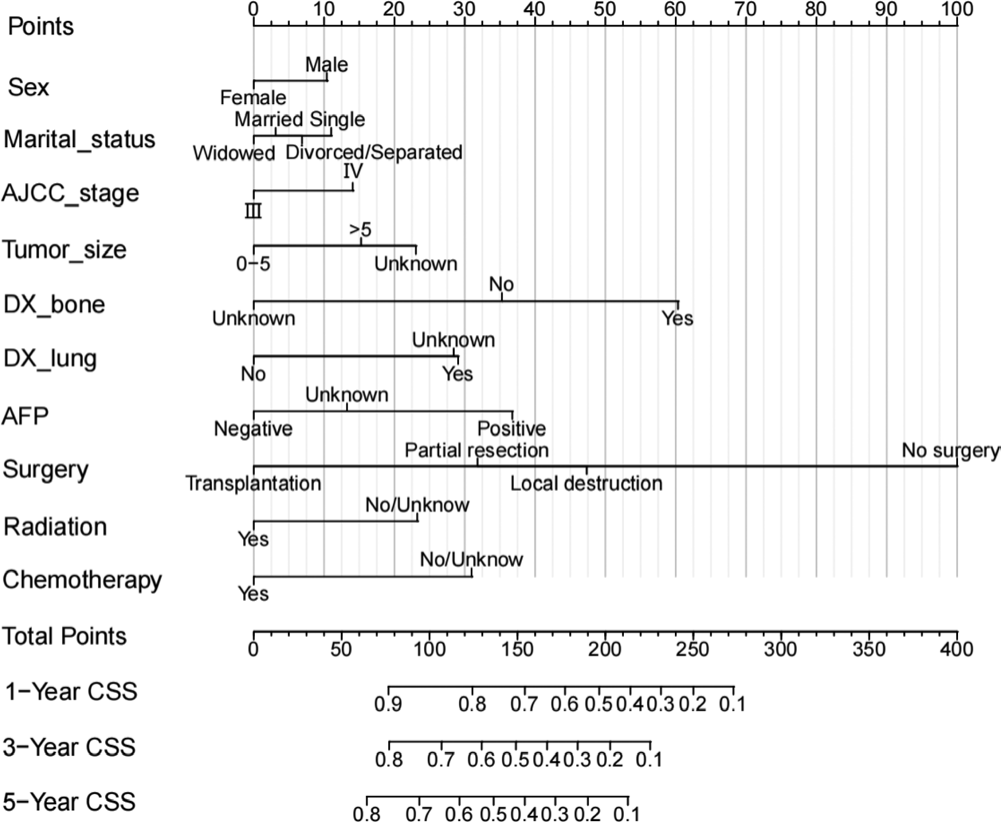
The nomogram of CSS in middle-aged patients with advanced HCC at 1-, 3-, and 5-year. AFP = alpha-fetoprotein, AJCC = American Joint Committee on Cancer, CSS = cancer-specific survival, HCC = hepatocellular carcinoma.

**Figure 3.**
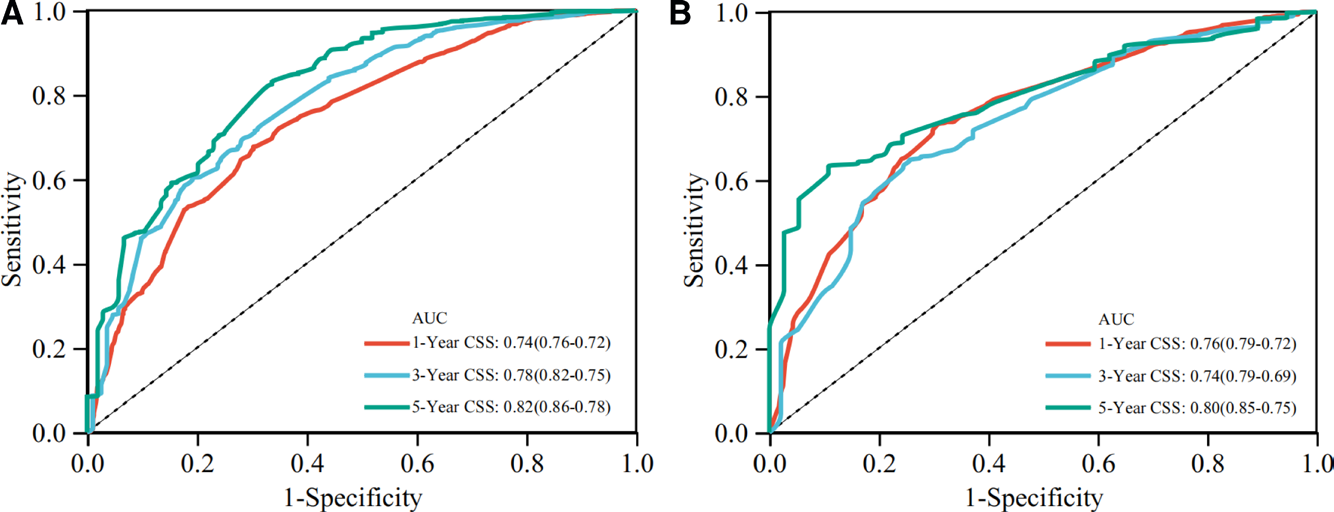
ROC of the nomogram in prediction of CSS at 1-, 3-, and 5-year. (A) Based on the training cohort; (B) Based on the validation cohort. AUC = area under the curve, CSS = cancer-specific survival, ROC = receiver operating characteristic.

**Figure 4.**
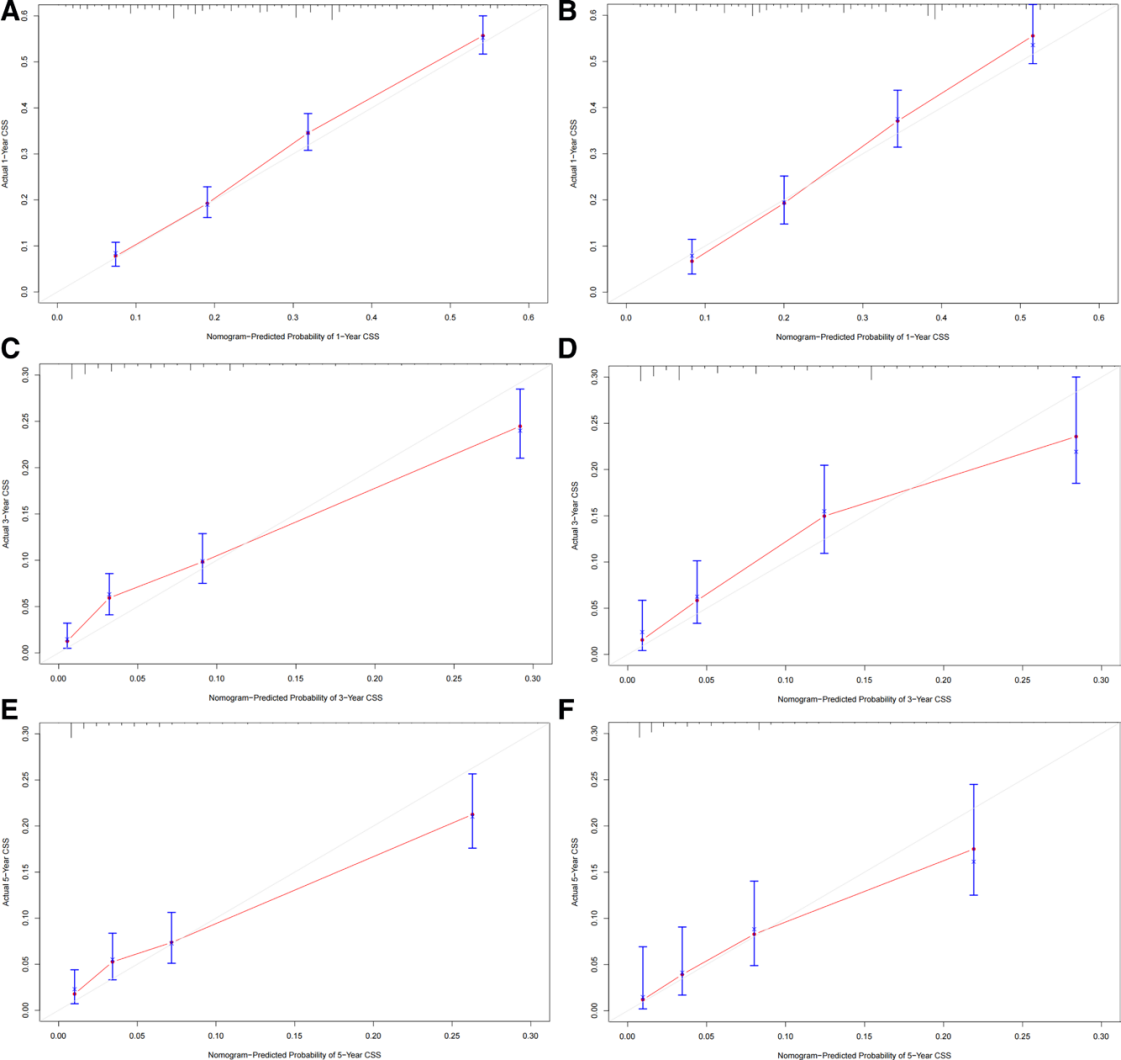
Calibration curves of the nomogram. (A, C, E) Calibration curves of 1-, 3-, and 5-year CSS in training cohort; (B, D, F) Calibration curves of 1-, 3-, and 5-year CSS in the validation cohort. CSS = cancer-specific survival.

**Figure 5.**
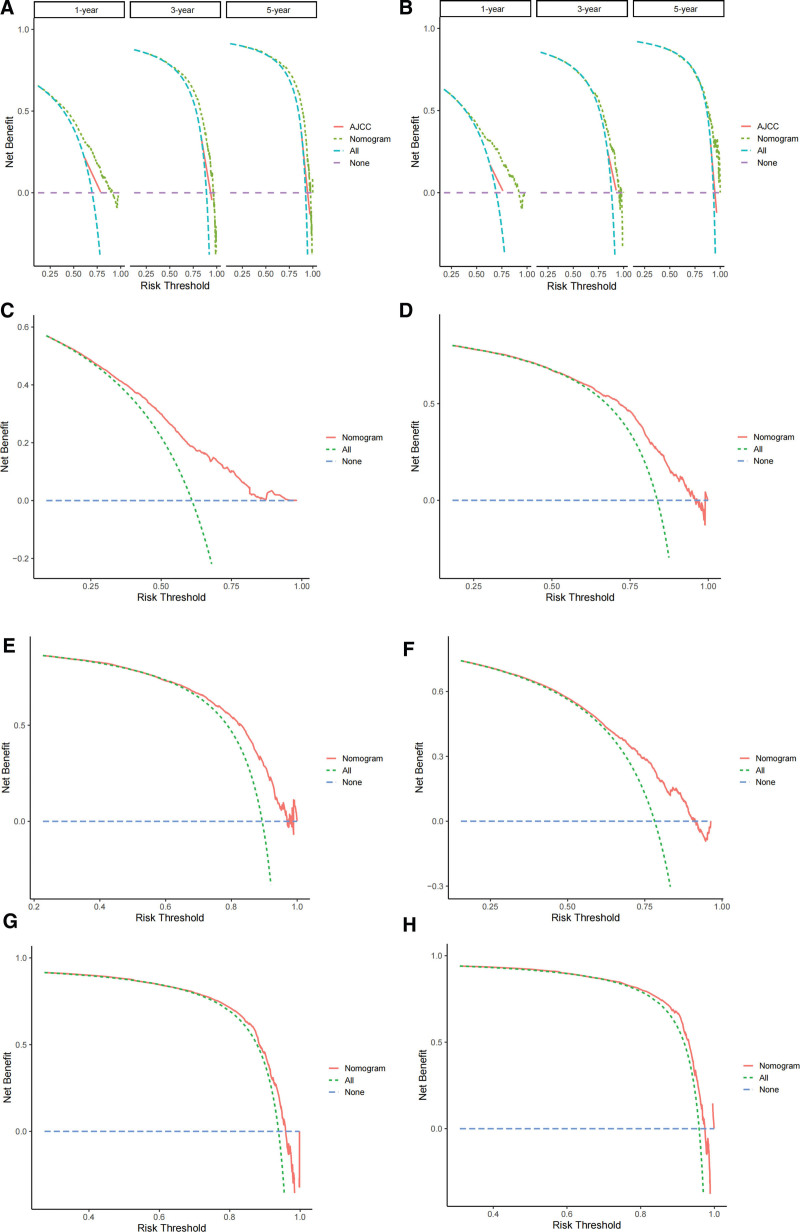
Decision curve analysis of the nomogram and AJCC staging system. (A) DCA of 1-, 3-, and 5-year CSS in training cohort; (B) DCA of 1-, 3-, and 5-year CSS in the validation cohort. (C–E) DCA of 1-, 3-, and 5-year CSS in AJCC stage III; (F–H) DCA of 1-, 3-, and 5-year CSS in AJCC stage IV. AJCC American Joint Committee on Cancer, CSS = cancer-specific survival, DCA = decision curve analysis.

Based on the independent prognostic factors derived from the above analysis, we constructed a nomogram model to predict the 1-, 3-, and 5-year CSS in middle-aged patients with advanced hepatocellular carcinoma (Fig. 2). As can be seen, surgery and bone metastasis were the 2 most important factors affecting patient survival, followed by AFP level, chemotherapy, lung metastasis, tumor size, and radiotherapy. In addition, AJCC stage, marital status, and gender also had an impact on patient prognosis. We validated the nomogram with a C-index of 0.700 (95% CI: 0.684–0.712) and 0.702 (95% CI: 0.682–0.722) for the training cohort and the validation cohort, respectively. The 1-, 3-, and 5-year AUCs were 0.74, 0.78, and 0.82 for the training cohort and 0.76, 0.74, and 0.80 for the validation cohort, respectively, and the receiver operating characteristic curves for both cohorts showed good discriminatory power of this prediction model (Fig. 3). In the training and validation cohorts, the calibration curves for the 1-year, 3-year and 5-year CSS showed a high degree of consistency between the actual observed values and the predicted values of the nomogram model (Fig. 4). In addition, based on the results of the DCA analysis, the nomogram showed greater positive net benefit in both the training and validation cohorts compared to the AJCC staging system. We validated this for patients with AJCC stage III and AJCC stage IV, respectively, and showed that the nomogram showed a significant positive net benefit across all staging (Fig. 5).

### 3.4. Risk stratification system

**Figure 6.**
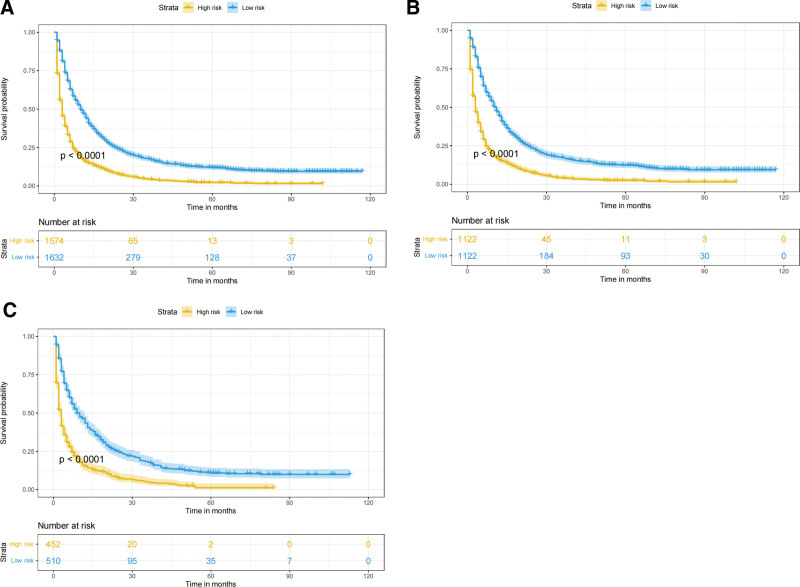
Kaplan–Meier curves of CSS for risk classification based on the nomogram scores. (A) In all cohort; (B) In the training cohort; (C) In the validation cohort. CSS = cancer-specific survival.

**Figure 7.**
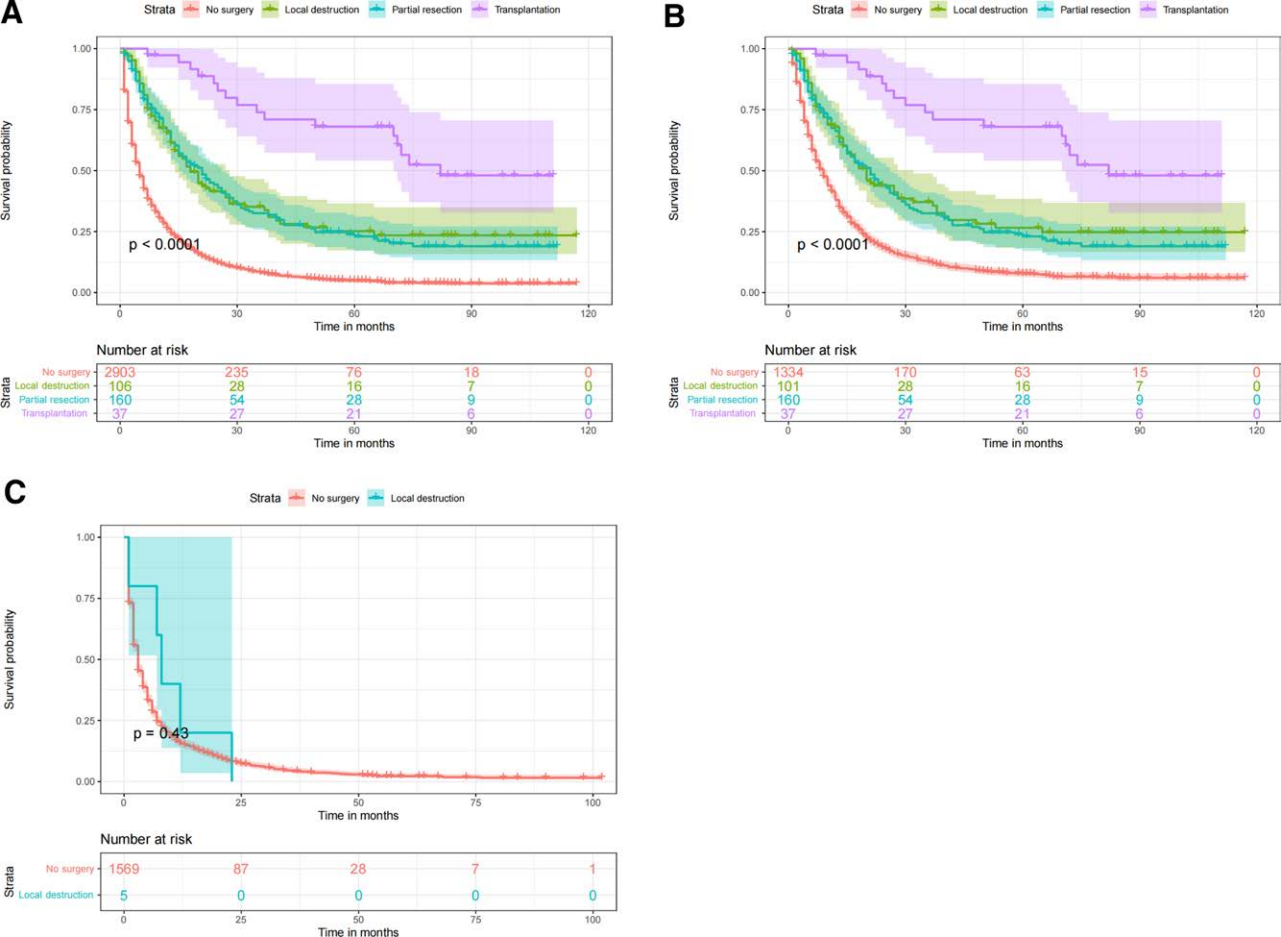
Kaplan–Meier curves of patients with different surgical procedures. (A) In all cohort; (B) In low-risk group; (C) In high-risk group.

All patients were divided into a low-risk group (total score ≤ 179.0) and a high-risk group (total score > 179.0) based on nomogram risk scores. Kaplan–Meier survival analysis showed that patients in the low-risk group had significantly higher CSS than the high-risk group in all cohort, the training cohort, and the validation cohort (*P* < .0001; Fig. 6). In addition, we compared the effects of different surgical procedures on survival in the low- and high-risk groups and in all cohort of patients. The results showed that in the low-risk group and in all cohort, patients who underwent liver transplantation, local destruction and partial resection had significantly higher survival rates than those who did not undergo surgery (*P* < .0001; Fig. 7A, B). In the high-risk group, there was no significant difference in survival rates between patients who underwent local destruction of the tumor and those who did not undergo surgery, which may be due to the small sample size of patients who underwent local destruction in this group (n = 5; Fig. 7C).

### 3.5. Web-based nomogram

**Figure 8.**
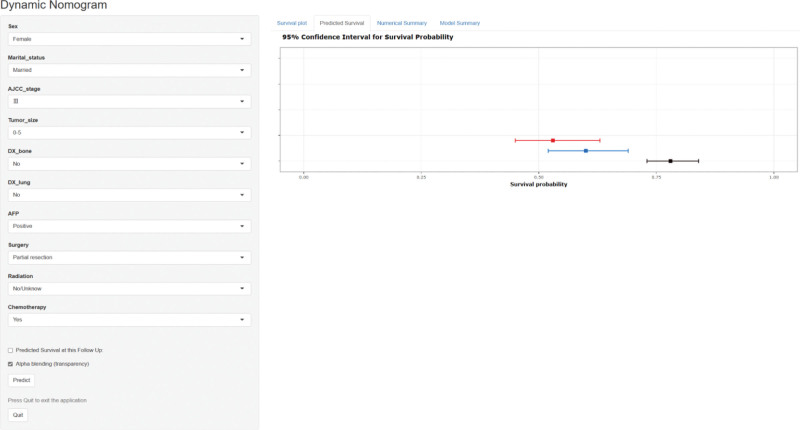
Screenshot of dynamic web-based nomogram.

**To facilitate clinical application, we have developed an easy-to-use, web-based dynamic nomogram that can be logged in from any electronic device. Clinicians and patients can quickly predict a patient’s CSS by directly inputting the appropriate independent prognostic factors. For instance, a 55-year-old AFP-positive married female hepatocellular carcinoma patient with AJCC stage III without bone metastasis, or lung metastasis underwent partial hepatectomy, and the postoperative pathology showed that the tumor size was 5 cm, and the patient was treated with chemotherapy after surgery. The patient’s CSS at 1-, 3-, and 5-years after receiving surgical treatment was 78%, 60%, and 53%, respectively. Using this site, a rapid prediction of the patient’s probability of survival can be achieved** (Fig. 8，https://nianliu.shinyapps.io/nomogram/).

## 4. Discussion

Advanced hepatocellular carcinoma was considered a difficult disease to treat in the chemotherapy era (1950–2000), and with the rise of systemic therapy, patient survival has improved slightly, but is still not promising.^[16,17]^ A number of nomogram models have been developed to assess the survival prognosis of HCC patients, identify high-risk patients, and implement individualized treatment to assist clinicians in making treatment decisions to improve patient survival. Yang et al^[18]^ constructed a nomogram model to predict CSS in patients with advanced HCC. Ni et al^[19]^ used the constructed nomogram to predict CSS and overall survival in patients with hepatocellular carcinoma. He et al^[20]^ developed a cancer-specific survival prediction model for elderly patients with early-stage hepatocellular carcinoma based on the SEER database. Wen et al^[5]^ also constructed and validated a nomogram to predict CSS in middle-aged patients with early-stage hepatocellular carcinoma.The importance of middle-aged people to family and socioeconomic development cannot be overstated, and they often assume important family and social roles. Based on clinical data from patients in the SEER database, this study was the first to target a study population of middle-aged patients with advanced hepatocellular carcinoma and developed a nomogram model and risk stratification system to predict their CSS at 1-, 3-, and 5-year, which has not been reported in other literature.

The construction of the nomogram was based on 10 independent risk factors (sex, marital status, AJCC stage, tumor size, bone metastasis, lung metastasis, AFP level, surgery, radiotherapy, chemotherapy) that we obtained from a multivariate Cox regression analysis that significantly influenced patients’ CSS. Using a risk score scale with the nomogram, we learned that surgery was the most important predictor of CSS. CSS was significantly worse in patients without any surgical treatment, which is consistent with the findings of He et al.^[20]^ HCC is more common in male patients and has a worse prognosis than in women. This may be due to the fact that men are more likely to drink alcohol and smoke, which are potential risk factors for several cancers, including HCC, with a relative risk of 2.07 for chronic drinkers compared to nondrinkers.^[21,22]^ In addition, there is an association with sex hormones.^[23]^ Our study found that CSS was longer in patients with tumor diameters of 0–5 cm than in those with > 5 cm, which is similar to the results of several other studies. Tumor size is considered an independent risk factor for prognosis in patients with HCC.^[24,25]^ Our previous study found that tumor size affected early recurrence in patients.^[26]^ Mortality was approximately 3-fold higher in patients with tumor diameter > 5 cm compared to patients with small tumors, and tumor diameter > 5 cm may be a good predictor of patient death and early recurrence when death is considered a competing risk.^[27]^ Patients with small tumor diameters (0–5 cm) tend to have more effective treatment opportunities such as surgical resection, liver transplantation and radiofrequency ablation, thus benefiting patient survival. AFP plays an important role in the malignant transformation of hepatocytes, regulating cell proliferation, migration and immune escape.^[28]^ High levels of serum AFP usually imply a high risk of development of hepatocellular carcinoma and a poor prognosis.^[29]^ In our study, a similar conclusion was derived that CSS was significantly shorter in AFP-positive patients than in AFP-negative patients. Furthermore, according to our findings, radiotherapy and chemotherapy improved the CSS of patients. The results of a prospective randomized controlled phase III clinical trial conducted by Lyu et al^[30]^ showed that interventional hepatic arterial chemotherapeutic drug infusion yielded a better benefit compared to sorafenib in the treatment of patients with advanced hepatocellular carcinoma. Another randomized open-label trial showed that sorafenib prolonged the median overall survival of patients, demonstrating good tumor control and safety, which confirms our conclusions.^[31]^ Although it remains controversial whether radiotherapy is effective in advanced hepatocellular carcinoma. However, it has also been shown that in patients with locally advanced hepatocellular carcinoma treated with selective internal radiotherapy, the objective remission rate at 3 months was significantly higher in the personalized dosimetry group compared to the standard dosimetry group.^[32]^

The C-index and AUC values of the training and validation cohorts showed great discriminatory ability of our constructed nomogram prediction model. The AJCC staging system along with the Barcelona Clinic Liver Cancer staging, Chinese liver cancer staging, and Japanese integrated staging score are several commonly used HCC staging systems nowadays.^[33–36]^ We compared the constructed nomogram with the AJCC staging system to determine whether the nomogram has potential for clinical application. The DCA results showed that the nomogram could obtain better positive net benefit compared with the AJCC staging system. **Subsequently, we compared patients with AJCC stage III and AJCC stage IV, respectively, and the results showed that the nomogram showed significant positive net benefit in both groups, and the nomogram had good predictive performance for patients with different staging stages, which further demonstrated the feasibility and practicality of our model.** In addition, in the new risk stratification system, Kaplan–Meier analysis showed that there was a highly significant difference in CSS between the low-risk and high-risk groups. This further demonstrates that the nomogram has the ability to discriminate between low and high risk groups better than the traditional staging system. This has important implications for clinicians’ treatment decisions and for providing individualized treatment plans.

Surgery is the treatment of choice for patients with early HCC and is currently the only way to achieve long-term survival or even a cure.^[37]^ Common surgical procedures include partial resection and liver transplantation. It is still controversial whether patients with advanced HCC should receive surgical treatment.^[38]^ Our findings suggest that surgery is the most important independent risk factor affecting CSS in middle-aged patients with advanced HCC. Kaplan–Meier analysis of the low-risk group and all cohort showed that liver transplantation was the most significant factor of the 3 surgical procedures affecting CSS compared to patients not treated surgically. Partial resection and local destruction also showed better survival outcomes. If conditions permit, we may perform surgery on eligible patients in the low-risk group after downstaging, conversion therapy, or other effective treatment for advanced HCC to prolong the CSS of patients as much as possible.

Although the nomogram showed good predictive performance, there are some limitations of our study. First, this study is a retrospective study based on the SEER database, and it is difficult to avoid bias, such as selection bias and recall bias. Secondly, the SEER database lacks some valuable clinical information, and patients’ serum biochemical indicators, **PIVKA II level (tumor marker), vascular invasion (tumor characteristics), Child-Pugh score,** HBsAg, HBV-DNA levels, and drinking history were not included. **Then, the nomogram we constructed limits the age range of patients to 40–60 years, which may not be applicable to other age groups, limiting its wide application in the population.** Finally, our model was only internally validated with the validation cohort, and more multicenter large-sample clinical data are needed for external validation. And the data were derived from the American population, which may not be representative of the rest of the world.

## 5. Conclusion

In conclusion, based on the large sample of clinical data provided by the SEER database, we developed a nomogram to predict CSS in middle-aged patients with advanced HCC. Compared with the AJCC staging system, the prediction model has great predictive performance and clinical utility, which helps clinicians make prognostic judgments and treatment decisions.

## Author contributions

**Conceptualization:** Ziqiang Li, Kun Li.

**Data curation:** Ziqiang Li, Qingyong Hong, Zhidong Guo, Xiaohong Liu, Chengpeng Tan, Zhe Feng.

**Formal analysis:** Ziqiang Li, Qingyong Hong, Chengpeng Tan.

**Funding acquisition:** Kun Li.

**Validation:** Ziqiang Li.

**Writing—original draft:** Ziqiang Li.
